# Medical students’ perceptions of a community-engaged learning approach to community health in Ghana: the Students’ Community Engagement Programme (SCEP)

**DOI:** 10.1186/s12909-024-06409-8

**Published:** 2024-11-30

**Authors:** Gifty Dufie Ampofo, Joseph Osarfo, Harry Kwami Tagbor

**Affiliations:** Department of Community Health, School of Medicine, University of Health and Allied Sciences, Ho, Ghana

**Keywords:** Community engaged learning, Community engagement, Medical students, Community health, Learning approach, Community interactions, Ghana

## Abstract

**Background:**

Revitalizing primary health care requires that the health workforce, especially doctors, must appreciate the significance of the socio-cultural environment in health. To achieve this objective, training of medical students must emphasize greater understanding of the community and its role in health through community engagement using community-engaged learning. However, research on this learning method applied in a purely community engagement context is lacking. A medical school in Ghana adapted its fourth-year junior clerkship curriculum in Community Health to include a community-engagement programme. This study reports students’ perceptions of the said programme as a way of evaluating it and helping to improve upon its implementation.

**Methods:**

A cross-sectional survey using a Google form-based questionnaire with open- and closed-ended questions was conducted from May 2022 to December 2023 among 303 current and past medical students of the University of Health and Allied Sciences, Ghana who had experienced the said programme. Based on the first three levels of the Kirkpatrick’s four-level model of training evaluation, data was collected on participants’ socio-demographics, their assessment of the programme content and delivery, subsequent attitudinal changes and their application to practice. Descriptive statistics were performed on quantitative data and thematic content analysis done for responses to the open-ended questions.

**Results:**

More than half of participants were males (188/303). The mean (SD) participant age was 23.9 years (2.4). Over 90% indicated the programme was valuable to their training (277/297) and were satisfied with the facilitators (283/297) despite some anxieties expressed at the start of their clerkship. Participants noted that they acquired other skills including teamwork and leadership aside the reinforced academic content. At least 99% of participants noted they could conduct the processes of community entry and engagement with or without assistance.

**Conclusion:**

Although there is room for improvement, the students’ community engagement programme may have some utility in the training of medical students to enable them better appreciate community interactions that influence health. Further research incorporating objective assessments of learning and behaviour change is needed to comprehensively assess the programme.

**Supplementary Information:**

The online version contains supplementary material available at 10.1186/s12909-024-06409-8.

## Background / context

Community-engaged learning (CEL) has been acclaimed as an effective teaching strategy in public health. It helps to acquire the knowledge, attitude and skill that medical students need to understand the balance between biologic and non-biologic determinants of health and to appreciate patients’ community contexts [1, 2]. This is best done in a framework of community engagement which entails developing relationships and working with groups of people with common interests in tackling health-related issues and promoting well-being to achieve desirable health outcomes [3].

This approach to learning offers useful opportunities for experiential learning which reinforces theoretical course content through community engagement and reflection on relevant activities [4, 5]. Ultimately, community-engaged learning contributes to shaping medical students for future roles as socially-responsive clinicians who can identify, assess and address broader sociocultural, economic and environmental determinants of health [1, 6, 7]. In seeking to strengthen primary health care and reform, the World Health Organization (WHO) contends that the health workforce, particularly medical doctors, should be aware of the significance of public health in the well-being of people and to use public health measures more in their interventions [8]. This point is further elaborated by calls for greater community engagement in any new orientation for training health professionals [9].

Though often linked with training in public health, CEL is used in many other disciplines including pharmacy practice, sociology education and engineering [10–12] and provides a platform for nurturing mutually beneficial relationships between academic institutions and community structures [5].

The goals of CEL are largely long term and this makes its evaluation challenging. This underpins the limited publications on the subject, especially in Africa [13]. Studies reporting evaluations of CEL programmes have broadly considered program design assessment [1, 5, 14], short-term learning outcomes based on student perceptions [12, 14, 15] as well as motivating factors and barriers to participation in such programmes [10]. Some evaluations of student health professionalcommunity engagement programmes looked at short-term objectives with a clinical bias and reported improvements in health knowledge, attitudes and practices among community members as well as varied student reflections on their experiences in the programme [10, 14, 16]. In a recent article exploring pharmacy students’ participation in CEL, some of their reflections hinted at contentment for the opportunity to develop their professions and careers and to positively impact the community while others lamented time constraints, the lack of confidence and preparatory information on the roles they were supposed to play in the context of activities constituting the community engagement programme [10]. Evaluation of clinically-oriented medical student clerkship programmes, solely involving interactions with health staff and patients in hospital settings in rural Australia and Canada, reported useful values in terms of team work and participatory learning among the students [17].

### The Students’ Community Engagement Programme (SCEP)

The University of Health and Allied Sciences (UHAS), Ho in the Volta Region of Ghana, West Africa, is a young public university established in 2012 with a vision to become a pre-eminent health research and practical-oriented health educational institution dedicated to community service. In line with this, innovative approaches to research, teaching and community engagement are encouraged. The Department of Community Health in the School of Medicine in UHAS, in its bid to innovate and make learning of Community Health beneficial to both medical students and surrounding communities, has proactively adopted a collaborative approach in engaging with these communities for teaching parts of the subject while contributing to improving their health status.

In UHAS, the fourth-year Junior Clerkship in Community Health is a 4-week programme designed for medical students to study the interactions and interrelations of health-related activities in the context of Primary Health Care at the community level. Students are to be exposed to health promotion and disease prevention activities and the health care delivery system in the community. They are expected to study in a practical way and apply theoretical knowledge acquired in specific Community Health concepts to analyse, understand and help solve health problems at the community level. The clerkship is intended to encourage students to function in teams and to develop their ability to collect relevant health-related information through observation and practical participation in health activities in the community. Students are also expected to develop the ability to critically assess and analyse this information and present it in the form of written reports and oral presentations at seminars for group discussion.

The fourth-year medical students’ Junior Clerkship in Community Health curriculum was adapted to implement a CEL programme dubbed the “Students’ Community Engagement Programme (SCEP)”. This derives from John Dewey’s learning-by-doing theory of education and emphasizes some principles including experiential learning, social learning and interaction, collaborative learning and critical reflection [18]. A cardinal principle in Dewey’s experiential learning theory is that human experience occurs through engaging a social environment with contact and communication [19]. Living and interacting in social environments such as the programme communities create a learning experience. Students reflect on these learning experiences to help construct knowledge which can then be transferred or applied to new situations [19]. These considerations underscore Dewey’s learning-by-doing theory as a theoretical lens to the current work. In agreement, other authorities have emphasized the multidisciplinary nature of education and that academic content needs to be complemented with learning activities in a variety of settings beyond the school [20, 21].

The SCEP concept seeks to build students’ capacity to undertake the following relevant public health-related activities; (i) community entry including using appropriate channels to reach community/traditional leadership and interacting with community members, (ii) community profiling and diagnosis including conducting a transect walk through the community, extracting information about the health of a community from health centre records, identifying and prioritizing health problems with community members, (iii) community mobilization and stimulated action including organizing durbars for sharing health information and liaising with existing organizations to undertake health promotion activities in the community, (iv) conducting epidemiological research including research problem identification and analysis, questionnaire development, data collection and basic data analysis and (v) reporting on activities and findings from the SCEP through writing, presentations and discussions. Overall, SCEP not only reinforces the curriculum but also contributes to students’ appreciation of community and socio-cultural contexts of illnesses.

To help achieve the objectives, the students spend up to two weeks living in designated, mostly rural and peri-urban, communities in near-by districts to build skills in community entry, profiling, diagnoses and community mobilisation/ engagement through interaction with community structures. In addition, based on their observations from the community diagnoses, they carry out health promotion and preventive activities by giving health talks in community durbars, schools and local radio stations Working together with community health nurses and volunteers, they also conduct health screening activities. Where possible, they initiate a sustainable health project having built consensus with the community.

Furthermore, the students conduct research into identified prevailing health issues in the community and use the opportunity to hone their questionnaire design / administration, data collection, organization, analysis and report writing skills. One such study assessed the water, sanitation and hygiene situation in ten communities across two districts [22]. About 5–15 students are sent to a community depending on the size of the community. Accommodation and, sometimes, feeding of these students are catered for mainly by the traditional leadership of the communities. Sometimes, health facilities in these communities may offer accommodation too.

Prior to leaving for their designated communities, the students have lectures on topics relevant to their stay in the community. These include the healthcare system in Ghana, health and the environment, community entry, engagement, mobilisation and diagnoses and health promotion. Building on the previous year’s introduction to research methodology course, the students are guided by their lecturers in identifying problems, setting objectives and in developing appropriate questionnaires from literature review on a common health-related issue. These questionnaires are administered to relevant participants in the communities to build the students’ competencies in data collection. On the field, they are aided and supervised by designated community preceptors who may be healthcare workers in the local health facilities or members of the community health committees. These preceptors are considered partners in the SCEP and they receive training from the Department of Community Health on their roles in the SCEP. While the students are in the communities, the faculty also visit them and provide some supervision of their activities as well. Indeed, an initial community entry is done by the faculty before the students get to their designated communities as a preliminary measure to ensure that accommodation and other support are available for the students, among other reasons.

Existing literature is lacking in research on CEL for medical students that is purely public health-oriented and engages with community members, their non-biologic health determinants and their belief systems. Many studies on the subject report on CEL with a bias for clinical orientation in local hospitals in much larger urban settings rather than emphasizing engagement with the community membership on health promotion and disease prevention activities [6, 14–16, 23–25].

Since its inception, six batches of students have passed through SCEP with the first four graduating from medical school already and working in various hospitals across the country. However, SCEP is yet to be evaluated with respect to the following research questions; (i) what are students reactions to or perceptions of the usefulness and processes of the programme?, (ii) what broad scope of knowledge and skillsets do students perceive they learnt or strengthened from SCEP, and (iii) whether students perceive that the knowledge and/or skills learnt have translated to behaviour change in attitude and practice towards patients? There is currently no data describing its successes or deficiencies and its perceived effects on learning have been anecdotal so far. This gap in knowledge is problematic as faculty have no insights at all into what participants in the programme (students and community members) perceive of the programme. There is no evidential basis to identify programme gaps or to inform appropriate interventions to address them. A need to conduct some assessment of the programme to help fill knowledge gaps was pertinent. Thus, an assessment of students’ perceptions of learning and training outcomes in Community Health through SCEP and community members’ perceptions of SCEP was conducted.

This report focuses on students’ perceptions and will contribute knowledge on the direct student-oriented short-term effects of the programme, inform on community-engaged learning, especially for medical students, in Ghana. It will also highlight weaknesses in the programme, draw suggestions on addressing them and guide didactic teaching of relevant topics including health promotion, community entry and participation and health priority setting.

## Methodology

### Study design and population

A cross-sectional survey, with self-administered questionnaires using Google forms, was conducted among the students. Since this was done online, no study site was involved. The study population comprised students of the School of Medicine, University of Health and Allied Sciences, Ho, Ghana who have passed through SCEP since its inception. Specifically, these were the various classes of 2020 to 2025 (the years representing when they graduated or will graduate from medical school).

### Sample size determination

No formal sample size calculation was required as all members of the study population totalling 377 (class sizes ranging from 41 to 72) were targeted for the survey. Table 1 shows the different participating classes and the number of students.

**Table 1:** Classes that participated in SCEP and their sizes

**Class**	**Number of students in the class**
2020	41
2021	55
2022	72
2023	72
2024	65
2025	72

### Study procedures and data collection tools

Data collection was done from May 2022 to December 2023. In line with global adoption of social media, students in institutions of higher education often create class WhatsApp groups and these maintain functionality long after they have graduated from school. Leveraging on this, the leaders of the various student classes were contacted and sent the online questionnaire for onward posting on their class WhatsApp platforms. An electronic mail was further sent to explain the purpose of the study to the classes that had graduated at the of time data collection while those still in school were met in person for the same purpose.

The questionnaire used for the survey (Supplementary file 1) was developed de novo for this study. It was based on students’ own perceptions of the first three levels of the Kirkpatrick’s Model [26]. The Kirkpatrick’s Four-Level Model of Training Evaluation [26] was originally used for evaluation of trainings in formal organizational settings but is applicable to evaluation of programmes in higher education [27]. The model emphasizes ‘reaction’, ‘learning’, ‘behaviour’ and ‘results/impact’ as the four levels representing a continuum of complexity in evaluating training programmes and other educational interventions [28]. In formal training settings, ‘reaction’ encompasses participants’ judgement of matters such as the training materials and content, delivery methods used, ability of the instructors to carry the participants along, convenience of the training venue, etc. The second level or ‘learning’ assesses the extent of attitudinal changes, knowledge improvement or skill acquisition as a result of participating in the training programme. The third level or ‘behaviour’ looks at how training-derived knowledge and skills are applied to one’s job routine post-training and emphasizes the point that merely acquiring new knowledge or skill does not translate directly into application. The fourth level looks at impact of the training on organizational goals and tends to be more of a medium-to-long term effect.

The Kirkpatrick’s Model [26] model has been criticized for the simplicity of its assumptions that the four levels are positively inter-correlated when there is no true guarantee that positive reactions (in level 1) will necessarily lead to good learning (level 2) and behavioural (level 3) outputs [29]. It however remains a useful framework for evaluating educational interventions / trainings [28].

The fourth level was excluded from the scope of this study as its evaluation needed to incorporate elements such as whether the programme influenced the career choices of the students or their location of practice. The participants were students still in school or relatively newly qualified doctors most of whom were still under apprenticeship as housemen. Evaluations pertaining to the ‘fourth level’ could thus not be included in this study.

The questionnaire had 5 sections. Section A was for socio-demographic data including age, sex and the current status of the student at the time of data collection. Section B addressed ‘reaction’ and personal reflection and included questions such as whether the students felt SCEP was worth their time, whether it was successful and whether they had any anxieties about the whole experience. The questions in section B had responses ‘no’, ‘yes’ and ‘not sure’. Section C was on ‘learning’ and focused on a list of competencies the students were expected to acquire during SCEP. The responses to these competencies were ‘can perform without assistance’, ‘can perform with assistance’ and ‘cannot perform’. In section D, the questions explored behaviour change and sought to find out whether the participants were currently using knowledge / skills they learnt during SCEP and whether the programme had led to any change in behaviour towards their patients or career. The responses in Section D were also ‘no’, ‘yes’ and ‘not sure’. Lastly, section E explored general perceptions about SCEP with open-ended questions.

### Data management and analysis

The Google forms, by default, generate an Excel spreadsheet which was retrieved and exported into Stata 16 (Stata Corp, College Station, TX, USA) for analysis. Descriptive statistics were performed for the close-ended questionnaires and frequencies, proportions and percentages presented in text and tables. The mean and standard deviation were also presented for the continuous variable ‘age’. Responses to open-ended questions were treated as qualitative data and inductive thematic content analysis done. Novel categories / themes were developed from the coding process and presented with supporting quotes. No pre-defined themes were used. Coding was done manually and independently by JO and GDA. Disagreements were resolved through discussion and consensus building with a focus on the study objectives.

### Ethical considerations

Ethical approval for the study was granted by the University of Health and Allied Sciences Research Ethics Committee with reference number UHAS-REC A.7 [12] 21–22. The Google form used explained that participation in the survey was voluntary and documented informed consent with the click of a button which was a required task before one could proceed further to the next parts of the questionnaire. Participants were anonymized by assigning them study identification numbers involving their matching serial numbers on the Excel spreadsheet generated from the Google forms. These serial numbers represent the order in which participants’ responses were automatically documented. Participants were also assured that the information they gave would only be accessible to faculty of the Community Health Department.

## Results

### Respondents’ background characteristics

Out of a potential pool of 377 respondents, a total of 303 (80.4%) filled the online questionnaires and same were analysed. Participation in the study was lowest among the Class of 2020 (21/41, 51.2%) and highest in the Class of 2022 where everyone, save one person, participated. The Class of 2021, 2023, 2024 and 2025 had participation of 72.7% (40/55), 79.2% (57/72), 83.1% (54/65) and 83.3% (60/72) respectively. Table 2 shows the background characteristics of the 303 study participants. Their ages ranged from 20–36 years with the mean age (SD) being 23.9 years (2.4). Males (62%) and those in the Class of 2022 were in the majority.

**Table 2:** Background characteristics of respondents

**Variable**	**Frequency (n)**	**%**
**Age (years) (*N* = 302)**		
20–24	224	74.2
≥ 25	78	25.8
Mean (SD)	23.9 (2.4)	
**Sex (*N* = 303)**		
Female	115	38.0
Male	188	62.0
**Graduating class of respondents (*N* = 303)**		
Class of 2020	21	6.9
Class of 2021	40	13.2
Class of 2022	71	23.4
Class of 2023	57	18.8
Class of 2024	54	17.8
Class of 2025	60	19.9

### Participants’ reactions and personal reflections about SCEP

Table 3 shows participants’ responses to close-ended questions on their reactions and personal reflections about SCEP. At least, 9 in 10 respondents thought SCEP was successful (277/297), worth their time (283/296) and were happy/satisfied with the lecturers/facilitators (including field preceptors) for SCEP (283/297). Close to 70% (203/294) admitted to having anxieties about the entire programme including the communities they were going to reside in before they went there while the remaining either did not have any anxiety or were not sure if they had any anxiety prior to moving into their designated communities. Of the 203 respondents who reported having anxieties before visiting their communities, 176 (86.7%) indicated their anxieties were alleviated once they settled in those communities. About 10% (32/297) were not satisfied with preparations towards the SCEP during their time.

**Table 3:** Participants’ responses to close-ended questions bordering on their reaction and personal reflections about SCEP

	Frequency	%
***Do you feel that SCEP was worth your time? (N* = *296)***		
No	2	0.7
Not sure	11	3.7
Yes	283	95.6
***Do you think that SCEP was successful? (N* = *297)***		
No	10	3.4
Not sure	10	3.4
Yes	277	93.3
***Did you have any anxieties before visiting the community? (N* = *294)***		
No	79	26.9
Not sure	12	4.1
Yes	203	69.0
***Did you like the community to which you were posted? (N* = *297)***		
No	23	7.7
Not sure	21	7.1
Yes	253	85.2
***Were your anxieties alleviated once you settled in the community? ( N* = *296)***		
No	24	8.1
Not applicable	72	24.3
Not sure	24	8.1
Yes	176	59.5
***Were the preparations (lectures, seminars, pep talks) prior to SCEP satisfactory? (n* = *297)***		
No	32	10.8
Not sure	32	7.7
Yes	242	81.5
***Were you happy with the lecturers/facilitators for SCEP? (N* = *297)***		
No	4	1.3
Not sure	10	3.4
Yes	283	95.3

### Themes emerging on participants’ anxiety prior to embarking on SCEP

#### Theme 1: General living conditions and security

Expectations of general living conditions including accommodation, sanitation and feeding came out as a common source of anxiety the students had before embarking on SCEP. Participants also expressed worry about security, availability of food and access to technology including internet services in those communities.

*“Housing and toilet facilities. Internet access including mobile phone network availability and safety / security”* (P11, Class of 2021)

*“….wondering how to keep up with eating for 10 days without any freezer available and no money to buy food everyday if that option was available to me”* (P83, Class of 2023)

#### Theme 2: Uncertainty about community reception, achieving goals and environmental issues

Other sources of anxiety for the students prior to departing for the communities were whether the community was going to be receptive and interact with them in a welcoming manner and also how they were going to deal with the problem of sandflies they had heard about in some of these communities. Uncertainty about achieving set objectives due to the limited time available for the programme and the inability to communicate in the local language also gave rise to pre-departure anxiety among the students.

*“I wasn’t sure about the reception we were going to receive…”* (P207, Class of 2024).

*“Whether I will be able to cope with the environment considering the fact that our predecessors told us about sandflies…and also whether the community members will accept us”* (P52, Class of 2023).

*“…not being able to finish data collection and not being able to identify a community problem that could be corrected”* (P37, Class of 2020).

*“…worried about the questionnaire administration since I could not communicate in the local dialect”* (P113, Class of 2022).

##### Theme 3: Role of group dynamics

The fragility of team formation and group dynamics appeared to be an additional element of anxiety that plagued the students prior to leaving for their designated communities.

*“….I was not comfortable with the teammates I had but I was proved wrong when we settled in the community….”* (P251, Class of 2025)

*“I wasn’t sure I could work with the people in my group because we were originally not close friends. I felt our level of understanding was very different and the work required collaboration and good teamwork”* (P161, Class of 2022).

### Emergent themes regarding alleviation of participants’ anxieties

The factors that helped to alleviate the students’ anxieties fell into three themes and came into play either before they embarked on the trip to their designated communities or when they arrived at the said communities.

#### Theme 1: Satisfactory living conditions and community receptions

Getting perceived satisfactory accommodation / living conditions in the community and good reception by local facilitators or preceptors and the community leadership went a long way to reduce participants’ anxiety. Quotes supporting this theme include the following;

*“The people provided good accommodation…I was able to get my food choices and we were supported by the community to undertake our project easily”* (P98, Class of 2020, Male).

*“…Good housing with water and electricity…affable preceptors, assemblyman and community leaders”* (P11, Class of 2021, Male)

#### Theme 2: Reassurance and adopting a coping attitude

Reassurances from their predecessors (students who had been to these communities earlier) and lecturers that they had nothing to worry about as well as adoption of a coping and adaptive mindset seemed to play a role in mitigating the students initial anxieties.

*“My anxieties were not necessarily alleviated. I just did what I had to do anyways”* (P25, Class of 2021, Female)

*“Acceptance of the sandflies because all methods to get rid of them did not work”* (P52, Class of 2023, Female)

*“…even though the washroom and bathroom were exactly what I feared it would be, I convinced myself that it was just 10 days and it will be over soon”* (P252, Class of 2025, Female).

*“Reassurance by our lecturers…hospitality and assistance by the townsfolks”* (P122, Class of 2022, Female)

### Themes emerging regarding the most important lessons students learnt during SCEP

Rather than just listing the two most important things learnt from their SCEP experience, the 286 participants who responded to this question gave nuanced contexts of the most important things they learnt over the period.

#### Theme 1: Academic content

Participants noted the practical reinforcement of the subject content of Community Health including community entry, community engagement, community diagnosis, and community mobilization and profiling. They appreciated how these subject matters played out in real life experiences to give them a better understanding of the concepts beyond what they had learnt in the classroom.

#### Theme 2: Soft skills

The students also emphasized learning a number of life lessons that have been collectively themed as ‘Soft skills’. Soft skills learnt entailed adaptability, leadership, empathy, teamwork and contentment. These covered a wide scope of lessons including the fact that one needs to be adaptive in life to achieve set goals as well as the need to appreciate the little comforts available to one.

*“Things don’t always go as planned but you have to find your way to achieve the goals you set…”* (P202, Class of 2024, Male)

*“…planning and leaving room for adaptation for better results….no matter the constraints that may come up…”* (P45, Class of 2023, Female)

*“….health needs to be targeted from the grassroots….it helped me to appreciate patients when they arrive in the consulting room”* (P110, Class of 2022, Male)

*“I learnt not to take things for granted as the people over there had issues with water (access) which I have barely had any issues with growing up”* (P109, Class of 2022, Female)

#### Theme 3: Improved appreciation of preventive medicine

Another key lesson learnt bordered on a better appreciation of the broader scope of medicine and medical practice beyond curative medicine to preventive medicine.

*“The life of a doctor is beyond the consulting room. Most of the diseases can be prevented.”* (P32, Class of 2021, Male)

### Themes on what participants plan to apply or are applying in their career from what they learnt during SCEP

The responses to this question were categorized into 2 themes and presented below. It must be noted that four respondents indicated they had learnt nothing from SCEP worthy of application in their careers.

#### Theme 1: Community mobilization

Community and/or workforce mobilization practiced by some members of the earlier classes at their current places of work and the teamwork they appreciate appear to have had their roots in SCEP.

*“I mobilize people all the time and I learned this from Tsito”* (one of the communities in the programme) (P2, Class of 2021, Male)

*“It is important to work in the community you find yourself in …really get involved…it can impact attendance and use of the health services you provide. It is easier if you are accepted and trusted in the community”* (P282, Class of 2025, Female)

*“Teamwork and respect for the role of the other care providers”* (P13, Class of 2021, Male)

#### Theme 2: Empathy and patient / community-centeredness

Majority of the respondents emphasized empathy for patients and a renewed understanding of recognizing their social circumstances in health care delivery. Additionally, the need to involve community members in identifying their health needs in a communal sense also stood out.

*“Treating the patients as individuals and not just diseases”* (P43, Class of 2023, Female)

*“I would be happy if my medical career would not just be a title but impact the community as I think this programme did”* (P55, Class of 2025, Female)

*“I pay more attention to the needs expressed by community members during community outreaches rather than imposing what I think is their need from a health worker’s perspective”* (P37, Class of 2020, Male)

### Self-reported SCEP competencies among study participants

The numbers and percentages of study participants who reported they are capable of specific competencies SCEP was designed to help them build are presented in Table 4. Less than half of respondents (138/303) indicated they could establish access to a community on their own while over 60% (190/300) believed they could extract and analyse information about the health of a community from health facility records without assistance. Similarly, close to 80% (236/301) of respondents indicated they could conduct interviews / group discussions with health staff and community members for health information and needs assessment working alone. Less than a third of participants reported they would require assistance to enable them give health talks to community members in person or on local radio stations.

**Table 4:** Participants’ responses regarding ability to perform SCEP competencies

**Community Health competence**	**Can perform without assistance n (%)**	**Can perform with assistance n (%)**	**Cannot perform n (%)**
**Community Entry**			
Establish access to a community	138 (45.7)	164 (54.3)	0
Use appropriate channel to reach chief/elders to seek permission	165 (54.6)	136 (45.1)	1 (0.3)
Interact with community members including chiefs/elders	167 (55.3)	135 (44.7)	0
Employ appropriate exit process	182 (60.3)	118 (39.0)	2 (0.7)
**Community Profiling And Diagnosis**			
Conduct transect walk through community and drawing of social map	230 (76.2)	72 (23.8)	0
Extract and analyse information about health of community from health facility records	190 (63.2)	110 (36.5)	1 (0.3)
Conduct interviews/group discussions with health staff and community members for health information and needs assessment	236 (78.4)	65 (21.6)	0
Consensus building—identify and prioritize topmost health problem with community members, agree and plan to execute project	180 (59.6)	121 (40.1)	1 (0.3)
Conduct a community survey	125 (41.5)	175 (58.2)	1 (0.3)
Write a report on SCEP activities and findings	207 (68.8)	94 (31.2)	0
Provide feedback on SCEP activities to community	205 (67.9)	97 (32.1)	0
**Community Mobilisation And Health Promotion**			
Mobilise community for communal labour activities	109 (36.2)	192 (63.8)	0
Liaise with existing NGOs and other organisations to undertake activities of interest to promote the health of the community	79 (26.2)	218 (72.2)	5 (1.6)
Give health talks to community members in person or on local radio stations	221 (72.2)	80 (26.5)	1 (0.3)
Conduct health screening exercises	141 (46.5)	161 (53.5)	0
Mobilize and donate relevant items to identified groups and institutions	153 (50.8)	147 (48.8)	1 (0.3)

### Self-reported changes in participants’ behaviour and perceptions towards their patients / medical careers influenced by SCEP

Approximately 72% (216/302) of respondents indicated they were using lessons learnt from the SCEP in their current levels or positions (see Table 5) while about 20% (61/302) could not give a definitive answer as to whether this was the case in their experience. At least 4 in 5 respondents felt the SCEP had somehow changed their behaviour and perceptions towards their patients and their medical careers (see Table 5).

**Table 5:** SCEP-attributable self-reported changes in participants’ behaviour and perceptions towards their patients and medical careers

	**Yes n (%)**	**No n (%)**	**Not sure n (%)**
Are you using any of what you learned during SCEP in your current level or position?	216 (71.5)	25 (8.3)	61 (20.2)
Has the training in SCEP led you to change any behaviour of yours towards your patients?	260 (86.1)	13 (4.3)	29 (9.6)
Has the training in SCEP led you to change any behaviour of yours towards your medical career?	270 (89.4)	16 (5.3)	23 (7.6)
Has the training in SCEP led you to change your perceptions about your patients?	270 (89.4)	9 (3.0)	23 (7.6)
Has the training in SCEP led you to change any perceptions of yours towards your medical career?	248 (82.4)	23 (7.6)	30 (10.0)
Are you confident to teach non-medical personnel (students/staff) in your team what you learnt from SCEP?	264 (87.7)	5 (1.7)	32 (10.6)

Analysing the responses to open-ended questions on SCEP’s influence on behaviour and perceptions towards patients / medical career, the changed behaviour reported could be summed up in two themes;

#### Theme 1: Greater cognisance of patients’ circumstances in their health

The thoughts expressed centred mostly on suggestions that the study participants now better understand patients as active participants in their own health care rather than as passive recipients of information from healthcare providers. This realization subsequently reflects on new approaches to taking patients’ social histories and management that take cognisance of their environments, beliefs and cultural systems.

*“I thought some patients were just not being responsible enough about their health and that of their children. I still think so. Some do disregard health information provided them but at least now I got to understand that sometimes it's not the fault of the individual, but some challenges are peculiar to some settlements and the various members of that settlement will have to work together to help curb them. So, in as much as the individuals want to do something, the people around them do indirectly affect their health”* (P168, Class of 2022, Male).

*“Patients should [not]be treated going by the books only: I am more mindful now of the effect of their social and physical environment on their health and not just focusing on trying to treat them by the books”* (P22, Class of 2021, Female).

*“I understand how communities affect one’s health seeking behaviour. It may be as a result of inadequate infrastructure, personnel or beliefs of the community. As a result, I understand and treat them with respect while acknowledging these challenges and beliefs and doing my best to educate them to undo stereotypical beliefs and misconceptions.”* (P284, Class of 2025, Female)

*“….Accommodating their ideas and working with them to achieve a common goal in terms of their health”* (P193, Class of 2024, Male).

#### Theme 2: Changed perceptions / attitudes

Among others, the SCEP experience appeared to have sparked a like for teamwork which wasn’t the case previously for some respondents. Changed attitudes towards patients generally and some relevant community health activities such as health promotion were also expressed.

“I’ve come to like working in a team which I used to not like so much” (P133, Class of 2022, Male)

*“Health promotion is really important and I learnt just how important it is which has changed my attitude towards it.”* (P207, Class of 2024, Female)

“The patients who come to us want us to feel their situation rather than just prescribing” (P174, Class of 2022, Male)

### What participants liked or did not like about the lecturers/facilitators including preceptors for SCEP

The participants expressed appreciation for the professionalism and commitment of both faculty and field supervisors (preceptors) towards the success of the SCEP programme. This commitment apparently paid off in other quarters such as reinforcing the students’ capacity to carry out their final-year research project works which happen to be a requirement for graduation. Favourable sentiments were also expressed regarding how their inputs into the planning and organization of SCEP were also accepted and taken on board. Of the 282 responses to this question, one respondent was uncertain whether he liked anything while another respondent was resolute that he did not like anything about the lecturers / facilitators for SCEP.

*“They were friendly, understanding and very practical in their approach. The lectures were specifically tailored to help us achieve our objectives. Preparing and administering questionnaires during that period was critical in preparing us for our thesis. The mistakes made helped us avoid certain headaches during the final year thesis”* (P11, Class of 2021, Male)

*“I like that most of them have practical examples of what we were likely to face. The lecturers also had a lot of experience in the field and not just book knowledge”* (P70, Class of 2021, Male)

*“I like the way the lecturers were ever ready to welcome our ideas and involved us in whatever we did in class and outside class”* (P91, Class of 2023, Male)

*“The nurse at Adaklu Have (one of the communities in the programme) actually devoted her whole time to us and made sure we were okay and comfortable. Our lecturer (Dr. Gifty) was always calling in to know how things were going and even visited us before the 7 days program ended”* (P146, Class of 2022, Male)

The majority of respondents who provided answers to the question of what they disliked about the lecturers /facilitators for SCEP indicated there was nothing they disliked. Few respondents reported dislike for the observation that some of the lecturers / facilitators were too strict, condescending, came in for lectures at unplanned times and did not take their time to explain some key subject contents. There were concerns that there was information overload in a rather short period of time and that lectures were long and boring. These concerns came mainly from respondents in the earlier classes. Others observed that some of the local field facilitators (preceptors) did not appear to know much about the programme and were thus handicapped on exactly what to do. Furthermore, a male from the Class of 2020 indicated that the programme appeared to have been done in a haste and unorganized manner.

*“Sometimes the lecturers acted as though we were supposed to know things that weren’t taught in class….especially when it came to data analysis. I feel like they were treating us as experts rather than as beginners”* (P70, Class of 2021, Male)

*“Instructions were not so clear and so we were not clear in our minds what the whole programme was about”* (P38, Class of 2020, Male)

“*There should have been more training on the gathering of the data and also how to write a report. We found it quite challenging in our approach to writing our report*.” (P163, Class of 2022, Male)

### Participants’ best and worst SCEP experiences and suggestions for improving the programme

The best experiences shared were mostly to do with community engagement for various activities including health talks, screening exercises, clean-up exercises and local tourism such as mountain hikes and visits to water falls in some of the communities visited. The worst experiences had to do with uncontrollable insect (sandfly) bites in a particular community, language barrier in some cases, accommodation and access to water and sanitation that were perceived to be sub-optimal, having to contend with unbearable heat for lack of electric fans in sleeping places and poor turn-up by the community members in some communities to some activities the study participants organized. In spite of these unfavourable experiences, 97.3% (293/301) of study participants thought the SCEP ought to be continued in UHAS-SOM while the rest indicated they were not sure if it should continue. No participant gave a definitive ‘No’ as a response to this question.

Some key suggestions made by the study participants to improve the programme included the following;

- A need for the faculty/organizers to make a conscious effort to improve the accommodation used to host students.
- Providing feedback on the research activity conducted and supporting the communities to carry out suggested projects to sustain the interest of community members in the programme.
- The rationale and objectives for the SCEP ought to be thoroughly explained to students and more training provided on data collection and report writing.
- More time should be dedicated to the SCEP in the academic calendar.

## Discussion

The study reports medical students’ perspectives of a Students’ Community Engagement Programme (SCEP), community-engaged learning (CEL) approach to Community Health that emphasizes community engagement and interactions, in a medical school in Ghana. To the best of the authors’ knowledge, this is the first such report that focuses purely on a community orientation as opposed to the clinical orientation that characterizes most research on CEL among medical students. The work highlights the students’ perceptions of the usefulness of the SCEP, its challenges and how best to make it more meaningful.

Over 90% of participants felt SCEP was successful and valuable to their personalities and medical career in spite of the observation that majority of them harboured different anxieties prior to leaving for their designated communities. Some of the SCEP-related anxiety appeared to derive from a feeling of inadequate preparedness or insecurity about their confidence for the activities they were to conduct in their communities despite the fact that they felt they had received sufficient training to enable them perform such activities. This observation may be likened to the ‘imposter phenomenon’ or ‘imposter syndrome’ which describes a self-doubt of one’s own skill or competence though one acknowledges receiving the needed academic content [30]. The phenomenon has been described previously in undergraduate, graduate and post-doctoral students who exhibited self-doubt and wondered why others have confidence in them when they themselves questioned their own self-confidence [12, 31–33]. Marked forms of the phenomenon have been linked to anxiety and distress [30, 32]. Being able to undertake their outlined activities with or without some level of assistance, once they settled in these communities, reinforces the narrative that they were adequately prepared for SCEP. To help mitigate the challenge of the “imposter syndrome” and the anxiety it may be associated with, it may be useful to reach out to colleague faculty who are clinical psychologists to help prepare the students mentally before they depart to their designated communities. This is important for the mental health of the students so that they can be in the best frame of mind to optimize this learning experience. In addition, field preceptors and community leadership must be guided/oriented in more innovative ways by the Department of Community Health to give all necessary support for the students’ activities.

The participants in the present study expressed satisfaction with how academic content played out in the community setting during their engagements with community leaders and members and how these interactions consolidated their theoretical knowledge. This exemplifies established notions that formal academic work needs to be complemented with learning activities outside the school / classroom for education to be meaningful for both learners and society as captured in Dewey’s learning-by-doing theory of education [19–21]. This viewpoint is so important that some undergraduate sociology students, in a study of what students say about CEL, lamented the lack of opportunities to apply the many theories and statistical models they had learnt in school and even went further to accuse their institution of perpetuating social inequality in this context [12]. Though research on CEL of the nature described in the current study is lacking, it has been observed that CEL generally leaves a worthy impression on student development and that participating students often benefit in other non-academic areas such as improving on their communication skills, collaboration and patience [34–36]. This observation was made in the present study as participants indicated they learnt to appreciate skills such as teamwork, leadership and adaptability in a unique and hands-on manner.

Some participants in the present study indicated they were not satisfied with preparations towards the SCEP during their time and further intimated that some preceptors did not appear to have a good grasp of what was to be done under the SCEP. These may have likely occurred in the early days of the SCEP as these observations mostly came from participants in the Class of 2020 and are understandable. The students were the first batch to have gone through the SCEP and were the ‘experimental’ group. As such, the SCEP was not fully operational and there were some bottlenecks that needed some improvement. Thus, subsequent years of the SCEP have seen some elements of improvement, albeit subconsciously, without necessarily documenting challenges to consciously improve upon the programme. Faculty of the Department of Community Health continue to engage with current student populations and field preceptors to work towards continuous quality improvement in SCEP. One of the recently instituted practices is to have student predecessors coming to share their experiences with the current class embarking on the SCEP. This has been helpful in alleviating the pre-departure anxiety the students experienced.

Though majority of students had their pre-departure anxieties alleviated after settling in their communities, some participants could not fully ‘give up’ theirs and lived through the period by temporarily adapting to the circumstances underscoring their anxiety. Many of such anxieties had to do with perceptions that the accommodation and sanitation facilities some participants had were less-than-optimal. Currently, the department relies on community leadership to host students. Expectedly, there will be differences in the accommodation provided.

Social anxiety may arise from negative perceptions about varying situations including transitioning to new environments. It may be classified as a normal emotional response and has been described by Nowland and Qualter [37] among children transitioning to new schools. In an exploration of factors influencing pharmacy students’ participation in CEL activities in Canada, Fang et al. [10] reported that some students raised concerns about their personal safety when assigned to neighbourhoods they deemed unsafe. In the Canadian study, unlike the present study, the students were not even living in these supposedly unsafe areas. The students in the present study only lived in the communities between 7 to 10 days. Hence, whatever stimuli that precipitated their anxiety could have been temporary and not much of a challenge. This is reinforced by the observation that majority of them had the anxiety alleviated when they immersed themselves in the community activities.

Between 46%-60% of the current study’s participants indicated they could perform the processes of community entry on their own. For the key elements of community profiling and diagnosis, this proportion was higher at 60%-78%. Community entry, profiling and diagnosis are key steps in engaging with communities and it would have been preferable to see all participants indicating they are able to undertake these steps without assistance. Nonetheless, the percentages shown remain impressive in the context that these steps, though needed for the participants’ optimal functioning as clinicians / future clinicians, are not exactly core to their clinical practice. Where these participating clinicians or medical students end up as public health managers in the future, they would likely have work colleagues and/or subordinates for whom these competencies are core and who would provide these managers with the relevant assistance. It is thus gratifying to note that practically all participants felt they could carry out the processes of community engagement with or without assistance. It may also be argued that this is a one-time experience, and that the university/department could consider a second phase of the SCEP for the students before they complete their training in order to improve/reinforce the acquisition of these competencies.

At least 80% of the participants were of the view that the SCEP had changed their perceptions and behaviour towards their patients and medical careers. These changes bordered mainly on a better appreciation of the external contexts of patients including their economic, social, cultural and environmental circumstances, how these impact on their health status and how clinical management of their disease conditions ought to take cognisance of these different dispensations. These observations bring to reality the role of CEL in helping to prepare medical students to be socially responsive and adaptive clinicians in the future [1, 2, 7].

On the basis of the fore discussions and the self-reported competencies indicated for the relevant public health activities, one may opine that SCEP has been useful. It must be noted, however, that the lack of objective assessments of learning and behaviour change outcomes limits making conclusions about the effectiveness of the programme. The major challenge, in the authors’ opinion, lies with consolidating and improving upon the apparent gains of SCEP. A key question is how we can ensure that the reported positive behavioural change of seeing patients as the social beings that they are remain a core attitude of the study participants’ practice? Secondly, while the academic content of the SCEP appears to be without problems, there are some challenges with operationalizing the community component. In this regard, two key recommendations made by the students will be taken on board immediately.

First, the Department of Community Health and the School of Medicine need to consider an extension of the time allotted for the SCEP to help curb some of the complaints made about feeling under pressure to complete questionnaire administration and other activities in a short time. Further considerations on this matter ought to include efforts to improve upon the living spaces the students occupy during their stay in these communities. Although this is being done in some regard with for example, providing some floor lining, mattresses for use in empty bed steads, drinking water and light bulbs in rooms without them, these may not be enough according to students’ perceptions and improvements are needed. Undertaking a qualitative study to further understand the needs and challenges of the students as they stay in the communities is needed. Faculty should also consider engaging students in the earlier years when related courses are being taught on the topic to be researched in the community. This will help reduce the pressure during the short time prior to going to the communities where the questionnaires are developed and may free more time for students to be in the communities. The Department of Community Health should plan to include the dissemination of research findings to participating communities in the SCEP to help foster an improved sense of partnership and responsiveness to the SCEP-related activities.

The strength of the study lies in its novelty and the availability of nuanced responses to open-ended questions to help contextualize quantitative findings. However, it did not include in-depth interviews or focus group discussions which would have allowed follow-up questions to some of the responses provided. This somehow limits the depth of contextualization of findings. Also, apart from the participants’ self-reports provided, the study did not assess and report other evidence, from actual observation, attesting to the attainment of higher levels (learning and behaviour change) of the Kirkpatrick’s model. This is acknowledged as a study limitation and calls for cautious interpretation of the study findings. Nevertheless, the study findings provide a useful evaluation, after 6 years of implementing SCEP and provides actionable points to improve upon it.

## Conclusion

Study participants thought SCEP was valuable to their training and praised it for giving them the opportunity to align didactic lectures with social reality. They further recommended it should be continued as a core part of the Community Health curriculum. Despite these, some operational challenges exist that faculty need to address expeditiously for its future improvement. Dissemination of research study findings to the community is recommended to help foster better institution-community relations. Though the study findings derive solely from student perceptions and self-reports and lack objective assessment of learning and behaviour, they may still suggest that SCEP has potential which can be harnessed over time to achieve its desired objectives. Further evaluation of the programme regarding its relevance to context, fidelity of implementation to design, and achieving objective measures of outcome at the individual and community levels is needed to make more concrete recommendations regarding the widespread adoption of SCEP by other medical schools. Also, further research is needed to assess whether and how SCEP has influenced course design / development as well as teaching in the host department. This knowledge will help optimize the programme content for its objectives.

## Supplementary Information

Supplementary Material 1. Survey Questionnaire.

Supplementary Material 2. NEW SCEP Dataset 3.

## Data Availability

Data is provided within the manuscript or supplementary information files.

## Abbreviations

CEL: Community-engaged learning
WHO: World Health Organization
SCEP: Students’ Community Engagement Programme
