# Supplementary material for: Medical students’ perceptions of a community-engaged learning approach to community health in Ghana: the Students’ Community Engagement Programme (SCEP)
Source: BMC Med Educ. 2024 Nov 30;24:1400. doi: 10.1186/s12909-024-06409-8 (PMC11607921; doi:10.1186/s12909-024-06409-8)
Supplement: Supplementary file 1 — Supplementary Material 1. Survey Questionnaire. [file 12909_2024_6409_MOESM1_ESM.docx]

**Appendix 1**

**Medical students’ assessment of their training and learning of community health through the Student Community Engagement Programme (SCEP)**

**Questionnaire for Medical Students**

This survey is restricted to ONLY past and present clinical medical students of the University of Health and Allied Sciences, Ho. It is being conducted by the Community Health Department (CHD) of the School of Medicine, UHAS. It aims to assess students’ perceptions of their training and learning of Community Health through the Students’ Community Engagement Programme (SCEP) to improve upon its implementation. As a past level 400 medical student of UHAS, your participation in this survey is voluntary but extremely important as you would be contributing immensely towards improvement of the programme for future training of students. There is no perceptible risk to your person as you participate in this survey. Your confidentiality is assured as your names will not be linked to any information that you provide and the information generated will be accessible to only staff of the CHD. Kindly take about 20 minutes of your time to fill out this questionnaire.

I consent to participate in this survey YES…………. NO……………..

|  | ***Please answer the following questions by ticking appropriate boxes and writing appropriate answers in the spaces provided.*** | | | | | | |
| --- | --- | --- | --- | --- | --- | --- | --- |
|  | **SECTION A: SOCIO-DEMOGRAPHICS** |  |  |  |  |  | |
| 1 | Age (years) |  |  |  |  |  | |
| 2 | Sex | Male |  | Female |  | |  |
| 3 | Current status | Level 400 |  | level 500 |  | |  |
|  |  | Level 600 |  | Houseman |  | |  |
| 4 | Number of days spent in the community: |  | | | | | |
| 5 | Name of community posted to: |  |  |  |  |  | |
|  | **SECTION B: REACTION AND PERSONAL REFLECTION** | **Yes** |  | **No** |  | **Not sure** | |
| 6 | Do you feel that SCEP was worth your time? |  |  |  |  |  | |
| 7 | Do you think that SCEP was successful? |  |  |  |  |  | |
| 8 | Did you have any anxieties before visiting the community? |  |  |  |  |  | |
| 9 | Did you like the community to which you were posted? |  |  |  |  |  | |
| 10 | Were your anxieties alleviated once you settled in the community? |  |  |  |  |  | |
| 11 | Were the preparations (lectures, seminars, pep talks) prior to SCEP satisfactory? |  |  |  |  |  | |
| 12 | Did the training in community engagement activities on the field accommodate your personal learning style? |  |  |  |  |  | |
| 13 | Were you happy with the lecturers/facilitators for SCEP? |  |  |  |  |  | |
| 14 | Did the training in SCEP meet your expectations? |  |  |  |  |  | |
| 15 | List two most important things you learnt during SCEP. |  | | | | | |
| 16 | From what you learned, what do you plan to apply or are applying in your career as a medical doctor? |  | | | | | |
|  | **SECTION C: LEARNING** |  |  |  |  |  | |
|  | ***The following are a list of competencies that you were expected to acquire. Grade your level of competence. Tick the appropriate box.*** | **Can perform without assistance** | **Can perform with assistance** | | **Cannot perform** | | |
|  | **Community Entry** |  |  |  |  |  | |
| 15 | Establish access to community |  |  |  |  |  | |
| 16 | Use appropriate channel to reach chief/elders to seek permission |  |  |  |  |  | |
| 17 | Interact with community members including chiefs and elders |  |  |  |  |  | |
| 18 | Employ appropriate exit process |  |  |  |  |  | |

|  | **Community profiling and diagnosis** | **Can perform without assistance** | | **Can perform with assistance** | | | | | **Cannot perform** | | |
| --- | --- | --- | --- | --- | --- | --- | --- | --- | --- | --- | --- |
| 19 | Conduct transect walk through community and drawing of social map |  | |  | | |  | |  |  | |
| 20 | Extract and analyse information about health of community from health centre records |  | |  | | |  | |  |  | |
| 21 | Conduct interviews and group discussions with health staff and community members for health information and needs assessment |  | |  | | |  | |  |  | |
| 22 | Consensus building- identify and prioritise topmost health problem with community members, agree and plan to execute project |  | |  | | |  | |  |  | |
| 23 | Conduct a community survey |  | |  | | |  | |  |  | |
| 24 | Write a report on SCEP activities and findings |  | |  | | |  | |  |  | |
| 25 | Provide feedback of SCEP activities to community |  | |  | | |  | |  |  | |
|  | **Community mobilisation and health promotion** |  | |  | | |  | |  |  | |
| 26 | Mobilise community for communal labour activities |  | |  | | |  | |  |  | |
| 27 | Liaise with existing NGO's and other organisations to undertake activities of interest to promote the health of community |  | |  | | |  | |  |  | |
| 28 | Give health talks to groups of community members/health talks on local radio stations |  | |  | | |  | |  |  | |
| 29 | Conduct health screening exercises |  | |  | | |  | |  |  | |
| 30 | Mobilise and donate relevant items to identified groups and institutions |  | |  | | |  | |  |  | |
| 31 | Work as an effective team member at the community level for health promotion and education |  | |  | | |  | |  |  | |
|  | **SECTION D: BEHAVIOUR** | **Yes** | |  | | | **No** | |  | **Not sure** | |
| 32 | Are you using any of what you learned during SCEP in your current position? |  | |  | | |  | |  |  | |
| 33 | Has the training in SCEP led you to change any behaviour of yours towards your patients? |  | |  | | |  | |  |  | |
| 34 | Has the training in SCEP lead you to change any behaviour of yours towards your medical carrier? |  | |  | | |  | |  |  | |
| 35 | Has the training in SCEP lead you to change any perceptions of yours about patients? |  | |  | | |  | |  |  | |
| 36 | Has the training in SCEP lead you to change any perceptions of yours towards your medical career? |  | |  | | |  | |  |  | |
| 37 | Are you confident to teach non-medical student members in your team what you learnt from SCEP? |  | |  | | |  | |  |  | |
| 38 | What behaviour of yours towards patients/medical career have you changed since undergoing the SCEP? |  | | | | | | | | | |
| 39 | What perceptions about patients/medical career have you changed since undergoing the SCEP? |  | | | | | | | | | |
|  | **SECTION E: GENERAL PERCEPTIONS ABOUT SCEP** |  | | | | | | | | | |
| 40 | What was your best experience with SCEP? |  | | | | | | | | | |
| 41 | What was your worst experience with SCEP? |  | | | | | | | | | |
| 42 | Do you think SCEP should continue in UHAS? | Yes | | | No | | | | Not sure | | |
| 43 | What would you suggest is done to improve upon SCEP? |  | | | | | | | | | |
| 44 | What is your overall level of satisfaction with SCEP? | Excel-lent | Very good | | | Good | | Fair | | | Poor |
| 45 | Any other comments? |  | |  | | |  | |  |  | |
